# Supplementary material for: SARS-CoV-2 disease severity and transmission efficiency is increased for airborne compared to fomite exposure in Syrian hamsters
Source: Nat Commun. 2021 Aug 17;12:4985. doi: 10.1038/s41467-021-25156-8 (PMC8371001; doi:10.1038/s41467-021-25156-8)
Supplement: Supplementary file 1 — Supplementary information [file 41467_2021_25156_MOESM1_ESM.docx]

**Supplementary information**

**SARS-CoV-2 disease severity and transmission efficiency is increased for airborne compared to fomite exposure in Syrian hamsters**

Julia R. Port^1*^, Claude Kwe Yinda^1*^, Irene Offei Owusu^1^, Myndi Holbrook^1^, Robert Fischer^1^, Trenton Bushmaker^1,2^, Victoria A. Avanzato^1^, Jonathan E. Schulz^1^, Craig Martens^3^, Neeltje van Doremalen^1^, Chad S. Clancy^4^, Vincent J. Munster^1#^

1. Laboratory of Virology, Division of Intramural Research, National Institutes of Health, Hamilton, MT, USA
2. Montana State University, Bozeman, Montana, USA
3. Rocky Mountain Genomics Core Facility, Division of Intramural Research, National Institutes of Health, Hamilton, MT, USA
4. Rocky Mountain Veterinary Branch, Division of Intramural Research, National Institutes of Health, Hamilton, MT, USA

*“These authors contributed equally to this work”

#Corresponding author: Vincent Munster, email: [vincent.munster@nih.gov](mailto:Vincent.munster@nih.gov)

**Supplementary Table 1:** RNA quantity and quality of lung samples for gene expression analysis. * marks low quality

| **Sample** | **Tissue weight (mg)** | **RNA conc. (ng/uL)** | **RNA yield (ug)** | **RIN** |
| --- | --- | --- | --- | --- |
| D1 aerosol | 65 | 99.9 | 8.0 | 2.7 |
| D1 aerosol | 44 | 35.4 | 2.8 | 2.4 |
| D1 aerosol | 61 | 20.8 | 1.7 | 2.1 |
| D1 aerosol* | 50 | 67.1 | 5.4 | 2.1 |
| D1 fomite | 15 | 16.7 | 1.2 | 3.7 |
| D1 fomite* | 40 | 6.0 | 0.4 | 5.9 |
| D1 fomite | 32 | 118.9 | 9.5 | 4.6 |
| D1 fomite | 7 | 54.6 | 4.4 | 4.4 |
| D1 IN | 22 | 222.6 | 17.8 | 6.1 |
| D1 IN | 8 | 173.5 | 13.9 | 5.8 |
| D1 IN | 11 | 38.4 | 3.1 | 7.1 |
| D1 IN | 9 | 66.6 | 5.3 | 5.1 |
| D4 aerosol | 60 | 18.5 | 1.3 | 3.5 |
| D4 aerosol | 53 | 65.1 | 5.2 | 3 |
| D4 aerosol | 56 | 112.6 | 9.0 | 2.6 |
| D4 aerosol | 57 | 91.2 | 7.3 | 3.2 |
| D4 fomite | 50 | 82.2 | 6.6 | 6.7 |
| D4 fomite | 40 | 29.7 | 2.4 | 5.5 |
| D4 fomite | 32 | 78.9 | 6.3 | 5.6 |
| D4 fomite | 32 | 87.1 | 7.0 | 7.5 |
| D4 IN | 11 | 126.6 | 10.1 | 7.3 |
| D4 IN | 3 | 52.7 | 4.2 | 6.3 |
| D4 IN | 15 | 117.7 | 9.4 | 6.6 |
| D4 IN | 15 | 80.8 | 6.5 | 7.6 |
| D1 control | 23 | 130.7 | 10.5 | 4.8 |
| D1 contro*l | 16.75 | ND | ND | ND |
| D1 control | 31.75 | 124.2 | 9.9 | 6.3 |
| D1 control | -8 | 19.4 | 1.6 | 5.8 |
| D4 control | 9.75 | 12.2 | 1.1 | 6.5 |
| D4 control | 12.75 | 55.3 | 4.4 | 5.9 |
| D4 control | 25.75 | 114.0 | 9.1 | 5.4 |
| D4 control | 42.75 | 106.6 | 8.5 | 6.6 |

**Supplementary Table 2:** Pathways differentially expressed in lungs collected at day 4 after inoculation with SARS-CoV-2. The data (significant genes) imported into Ingenuity Pathway Analysis was multiple test corrected using the false discovery rate (Benjamini Hochberg 1995).  The p-value for each canonical pathway was calculated by the right-tailed Fisher's Exact Test.

| **I.N. - Control** | | | **Aerosol - Control** | | | **Fomite - Control** | | | | |
| --- | --- | --- | --- | --- | --- | --- | --- | --- | --- | --- |
| **Ingenuity Canonical Pathways** | **-log(p-value)** | **z-score** | **Ingenuity Canonical Pathways** | **-log(p-value)** | **z-score** | **Ingenuity Canonical Pathways** | | **-log(p-value)** | | **z-score** |
| PI3K/AKT Signaling | 11.5 | 2.137 | EIF2 Signaling | 17.6 | 2.734 | Interferon Signaling | | 6.97 | | 2.121 |
| Fcγ Receptor-mediated Phagocytosis in Macrophages and Monocytes | 11.5 | 3.452 | Tec Kinase Signaling | 14.5 | 2.101 | Role of Hypercytokinemia/hyperchemokinemia in the Pathogenesis of Influenza | | 5.79 | | 3.162 |
| Tec Kinase Signaling | 10.5 | 2.967 | Glioblastoma Multiforme Signaling | 11.7 | -2.566 | Systemic Lupus Erythematosus In B Cell Signaling Pathway | | 2.64 | | 2.309 |
| Integrin Signaling | 10.2 | 2.06 | PI3K/AKT Signaling | 11.6 | 2.38 | Th17 Activation Pathway | | 2.39 | | 2.449 |
| Systemic Lupus Erythematosus In B Cell Signaling Pathway | 10.1 | 4.303 | Aldosterone Signaling in Epithelial Cells | 11.4 | -2.402 | Death Receptor Signaling | | 2.36 | | 2.449 |
| iNOS Signaling | 9.25 | 3.9 | Sphingosine-1-phosphate Signaling | 11.3 | -2.774 | Role of Pattern Recognition Receptors in Recognition of Bacteria and Viruses | | 1.83 | | 2.449 |
| Production of Nitric Oxide and Reactive Oxygen Species in Macrophages | 8.96 | 3.753 | Fcγ Receptor-mediated Phagocytosis in Macrophages and Monocytes | 10.1 | 3.175 | Retinoic acid Mediated Apoptosis Signaling | | 1.75 | | 2 |
| iCOS-iCOSL Signaling in T Helper Cells | 7.89 | 3 | Coronavirus Pathogenesis Pathway | 8.76 | -3.906 | Cell Cycle: G1/S Checkpoint Regulation | | 1.6 | | 2 |
| Type I Diabetes Mellitus Signaling | 7.89 | 4.017 | Factors Promoting Cardiogenesis in Vertebrates | 8.31 | -2.945 | Growth Hormone Signaling | | 1.52 | | -2 |
| Th1 Pathway | 7.89 | 4.116 | Cardiac Hypertrophy Signaling | 8.29 | -2.065 | Sphingosine-1-phosphate Signaling | | 1.34 | | -2 |
| Role of NFAT in Regulation of the Immune Response | 7.76 | 2.92 | CXCR4 Signaling | 8.2 | -2.06 |  | |  | |  |
| IL-6 Signaling | 7.38 | 3.333 | Gαq Signaling | 7.81 | -2.38 |  | |  | |  |
| CD28 Signaling in T Helper Cells | 7.36 | 2.785 | Systemic Lupus Erythematosus In B Cell Signaling Pathway | 7.6 | 4.322 |  | |  | |  |
| Signaling by Rho Family GTPases | 7.15 | 2.359 | iNOS Signaling | 7.59 | 3.962 |  | |  | |  |
| NF-κB Signaling | 7.02 | 2.064 | Relaxin Signaling | 7.32 | -3.651 |  | |  | |  |
| Interferon Signaling | 7.01 | 2.5 | Death Receptor Signaling | 6.66 | 2.469 |  | |  | |  |
| ERK/MAPK Signaling | 7.01 | 2.832 | Th1 Pathway | 6.57 | 2.714 |  | |  | |  |
| Natural Killer Cell Signaling | 6.54 | 3.753 | Wnt/Ca+ pathway | 6.15 | -2.694 |  | |  | |  |
| B Cell Receptor Signaling | 6.5 | 3.727 | Natural Killer Cell Signaling | 6 | 3.771 |  | |  | |  |
| Tumor Microenvironment Pathway | 6.39 | 3.015 | Kinetochore Metaphase Signaling Pathway | 5.78 | 2.137 |  | |  | |  |
| PI3K Signaling in B Lymphocytes | 6.27 | 2.744 | CREB Signaling in Neurons | 5.63 | -3.004 |  | |  | |  |
| Actin Cytoskeleton Signaling | 6.13 | 2.897 | IL-1 Signaling | 5.25 | 2.524 |  | |  | |  |
| Th2 Pathway | 5.97 | 2.959 | Apelin Cardiomyocyte Signaling Pathway | 5.18 | -2.03 |  | |  | |  |
| Factors Promoting Cardiogenesis in Vertebrates | 5.76 | -2 | Huntington's Disease Signaling | 5.17 | -2.887 |  | |  | |  |
| PDGF Signaling | 5.64 | 2.746 | TNFR1 Signaling | 5.08 | 3.266 |  | |  | |  |
| Rac Signaling | 5.38 | 2.268 | GNRH Signaling | 4.95 | -2.496 |  | |  | |  |
| HIF1α Signaling | 5.25 | 2.359 | Dendritic Cell Maturation | 4.93 | 2.433 |  | |  | |  |
| IL-15 Signaling | 5.18 | 2.711 | Type I Diabetes Mellitus Signaling | 4.93 | 4.459 |  | |  | |  |
| Role of NANOG in Mammalian Embryonic Stem Cell Pluripotency | 5.09 | 2.138 | Breast Cancer Regulation by Stathmin1 | 4.43 | -2.601 |  | |  | |  |
| TNFR2 Signaling | 4.98 | 2.887 | Cholecystokinin/Gastrin-mediated Signaling | 4.42 | -2.343 |  | |  | |  |
| Antiproliferative Role of TOB in T Cell Signaling | 4.89 | -2.138 | PCP pathway | 4.37 | -2.887 |  | |  | |  |
| Dendritic Cell Maturation | 4.67 | 3.569 | Erythropoietin Signaling Pathway | 4.33 | -2.582 |  | |  | |  |
| Role of Pattern Recognition Receptors in Recognition of Bacteria and Viruses | 4.65 | 3.4 | Neuroinflammation Signaling Pathway | 4.33 | 4.013 |  | |  | |  |
| Pancreatic Adenocarcinoma Signaling | 4.54 | 2.985 | Cell Cycle Control of Chromosomal Replication | 4.03 | 3.4 |  | |  | |  |
| IL-2 Signaling | 4.49 | 2 | Toll-like Receptor Signaling | 3.93 | 2.524 |  | |  | |  |
| Synaptogenesis Signaling Pathway | 4.4 | 2.213 | B Cell Receptor Signaling | 3.89 | 2.689 |  | |  | |  |
| Crosstalk between Dendritic Cells and Natural Killer Cells | 4.34 | 3.9 | CD28 Signaling in T Helper Cells | 3.87 | 2.137 |  | |  | |  |
| Thrombopoietin Signaling | 4.28 | 2.524 | Role of Pattern Recognition Receptors in Recognition of Bacteria and Viruses | 3.8 | 2.466 |  | |  | |  |
| Type II Diabetes Mellitus Signaling | 4.27 | 2.353 | tRNA Charging | 3.8 | 3.441 |  | |  | |  |
| EGF Signaling | 4.04 | 2.183 | Unfolded protein response | 3.56 | 3.545 |  | |  | |  |
| TNFR1 Signaling | 4.03 | 3.357 | Interferon Signaling | 3.26 | 3 |  | |  | |  |
| 4-1BB Signaling in T Lymphocytes | 3.9 | 2.121 | IL-15 Signaling | 3.25 | 2.043 |  | |  | |  |
| Role of PKR in Interferon Induction and Antiviral Response | 3.88 | 2.502 | TREM1 Signaling | 2.88 | 4.158 |  | |  | |  |
| Acute Phase Response Signaling | 3.85 | 2.197 | iCOS-iCOSL Signaling in T Helper Cells | 2.86 | 2.414 |  | |  | |  |
| PKCθ Signaling in T Lymphocytes | 3.85 | 4.243 | Basal Cell Carcinoma Signaling | 2.83 | -2.837 |  | |  | |  |
| Neuroinflammation Signaling Pathway | 3.79 | 4.901 | Endoplasmic Reticulum Stress Pathway | 2.73 | 3.317 |  | |  | |  |
| TREM1 Signaling | 3.65 | 2.683 | TNFR2 Signaling | 2.71 | 2.309 |  | |  | |  |
| Role of RIG1-like Receptors in Antiviral Innate Immunity | 3.57 | 2.333 | Tumoricidal Function of Hepatic Natural Killer Cells | 2.71 | 2.828 |  | |  | |  |
| PCP pathway | 3.53 | -2.5 | PKCθ Signaling in T Lymphocytes | 2.65 | 2.655 |  | |  | |  |
| p38 MAPK Signaling | 3.49 | 3.545 | Granzyme B Signaling | 2.59 | 2.121 |  | |  | |  |
| IL-1 Signaling | 3.23 | 2.84 | Purine Nucleotides De Novo Biosynthesis II | 2.51 | 2.646 |  | |  | |  |
| PPAR Signaling | 3.16 | -3.266 | Dopamine Receptor Signaling | 2.35 | -2.333 |  | |  | |  |
| Regulation Of The Epithelial Mesenchymal Transition By Growth Factors Pathway | 3.15 | 3 | Apelin Liver Signaling Pathway | 2.35 | -2.309 |  | |  | |  |
| Renin-Angiotensin Signaling | 3.12 | 2.041 | Valine Degradation I | 2.34 | -2.828 |  | |  | |  |
| TCA Cycle II (Eukaryotic) | 3.05 | -3 | Cell Cycle: G2/M DNA Damage Checkpoint Regulation | 2.34 | -2.324 |  | |  | |  |
| Valine Degradation I | 2.98 | -2.646 | Antiproliferative Role of TOB in T Cell Signaling | 2.32 | -2.84 |  | |  | |  |
| B Cell Activating Factor Signaling | 2.78 | 2.121 | Cdc42 Signaling | 2.32 | 2.271 |  | |  | |  |
| Ceramide Biosynthesis | 2.67 | -2 | VDR/RXR Activation | 2.27 | 2.183 |  | |  | |  |
| Th17 Activation Pathway | 2.52 | 3.578 | Crosstalk between Dendritic Cells and Natural Killer Cells | 2.27 | 4.796 |  | |  | |  |
| Necroptosis Signaling Pathway | 2.47 | 2.556 | Superpathway of Serine and Glycine Biosynthesis I | 2.18 | 2.236 |  | |  | |  |
| Role of Hypercytokinemia/hyperchemokinemia in the Pathogenesis of Influenza | 2.45 | 4.359 | dTMP De Novo Biosynthesis | 2.05 | 2 |  | |  | |  |
| Telomerase Signaling | 2.34 | 2.524 | Folate Polyglutamylation | 2.05 | 2 |  | |  | |  |
| Basal Cell Carcinoma Signaling | 2.17 | -2.333 | IL-17 Signaling | 1.96 | 2.449 |  | |  | |  |
| Oncostatin M Signaling | 2.11 | 2.714 | RAN Signaling | 1.77 | 2.121 |  | |  | |  |
| Isoleucine Degradation I | 2.04 | -2.236 | LXR/RXR Activation | 1.67 | -2.556 |  | |  | |  |
| CNTF Signaling | 1.95 | 2.496 | Purine Nucleotides Degradation II (Aerobic) | 1.45 | 2.828 |  | |  | |  |
| Cytotoxic T Lymphocyte-mediated Apoptosis of Target Cells | 1.91 | 2.121 | Aspartate Degradation II | 1.37 | 2 |  | |  | |  |
| MIF-mediated Glucocorticoid Regulation | 1.91 | 2.333 | Guanosine Nucleotides Degradation III | 1.37 | 2.449 |  | |  | |  |
| Cell Cycle Regulation by BTG Family Proteins | 1.67 | 2 | Th17 Activation Pathway | 1.37 | 2.746 |  | |  | |  |
| Role of IL-17F in Allergic Inflammatory Airway Diseases | 1.67 | 2.333 | Gαs Signaling | 1.36 | -2.041 |  | |  | |  |
| IL-17 Signaling | 1.65 | 3.053 | Cytotoxic T Lymphocyte-mediated Apoptosis of Target Cells | 1.33 | 3.317 |  | |  | |  |
| Fatty Acid β-oxidation I | 1.53 | -2.828 |  |  |  | |  | |  |  |
| Hypoxia Signaling in the Cardiovascular System | 1.39 | 2.646 |  |  |  | |  | |  |  |
| Apelin Adipocyte Signaling Pathway | 1.35 | -2.138 |  |  |  | |  | |  |  |
| Tumoricidal Function of Hepatic Natural Killer Cells | 1.3 | 2 |  |  |  | |  | |  |  |

**Supplementary Table 3: qRT-PCR primers and probes for SARS-CoV-2**

1. **Subgenomic viral RNA qRT-PCR**

| Primer | Direction | Seq 5’-3’ |
| --- | --- | --- |
| sgLeadSARS2-F | F | CGATCTCTTGTAGATCTGTTCTC |
| E_Sarbeco_R2 | R | ATATTGCAGCAGTACGCACACA |
| E_Sarbeco_P1 | P | FAM-ACACTAGCCATCCTTACTGCGCTTCG-ZEN-IBHQ |

1. **Genomic qRT-PCR**

| Primer | Direction | Seq 5’-3’ |
| --- | --- | --- |
| E_Sarbeco_F1 | F | AACAGGTACGTTAATAGTTAATAGCGT |
| E_Sarbeco_R2 | R | ATATTGCAGCAGTACGCACACA |
| E_Sarbeco_P1 | P | FAM-ACACTAGCCATCCTTACTGCGCTTCG-ZEN-IBHQ |

**
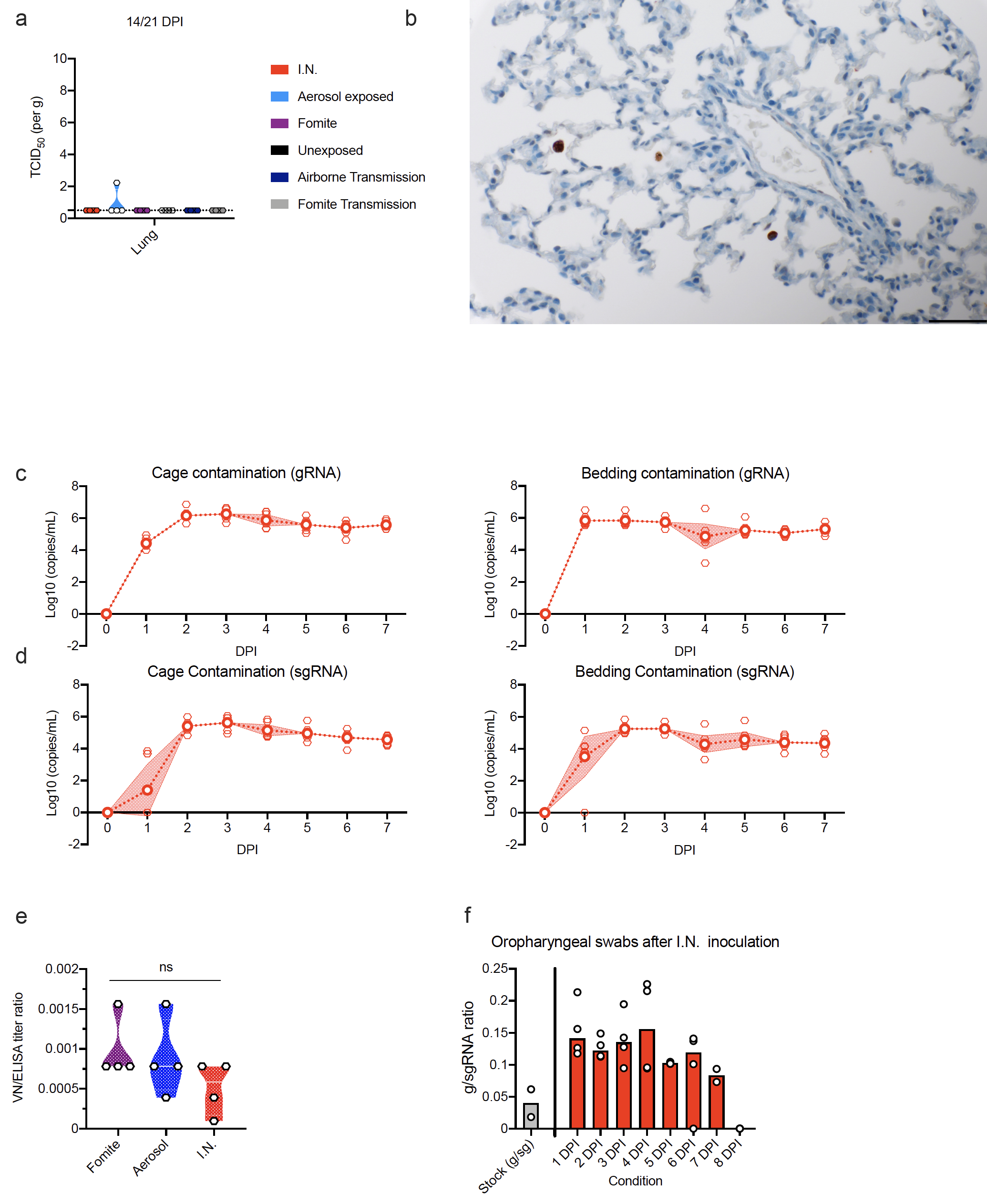
**

**Supplementary Figure 1: a.** Violin plot of infectious SARS-CoV-2 titer in the lungs of all animals at 14- or 21-days post inoculation or exposure (DPI). I.N. = intranasal**. b.** Day 1 intranasal immunohistochemistry (N = 4). Diffusely throughout all evaluated lung lobes are low to moderate numbers of macrophages showing cytoplasmic immunoreactivity to SARS-CoV-2 N protein. Adjacent alveoli (lower right) contain individual pulmonary macrophages that are not immunoreactive to SARS-CoV-2. (IHC, 400x, bar = 50 μm). **c.** cage and **d.** bedding contamination by infected animals till 7 DPI. Median, 95% CI and individuals are shown. **e.** Violin plots with individuals and median of the ratio of endpoint IgG antibody titres against SARS-CoV-2 spike ectodomain measured by ELISA in serum and reciprocal live virus neutralization titers. Statistical significance was measured using a Kruskal-Wallis test **f.** Ratio of genomic to subgenomic RNA measured in inoculum stock of SARS-CoV-2 (grey (N = 2)) and oropharyngeal swabs of I.N. inoculated hamsters (red, N = 4). *P < 0.05, **P < 0.001, ***P < 0.0001, ****P < 0.0001. NS, not significant. Source data are provided as a Source Data file.

**
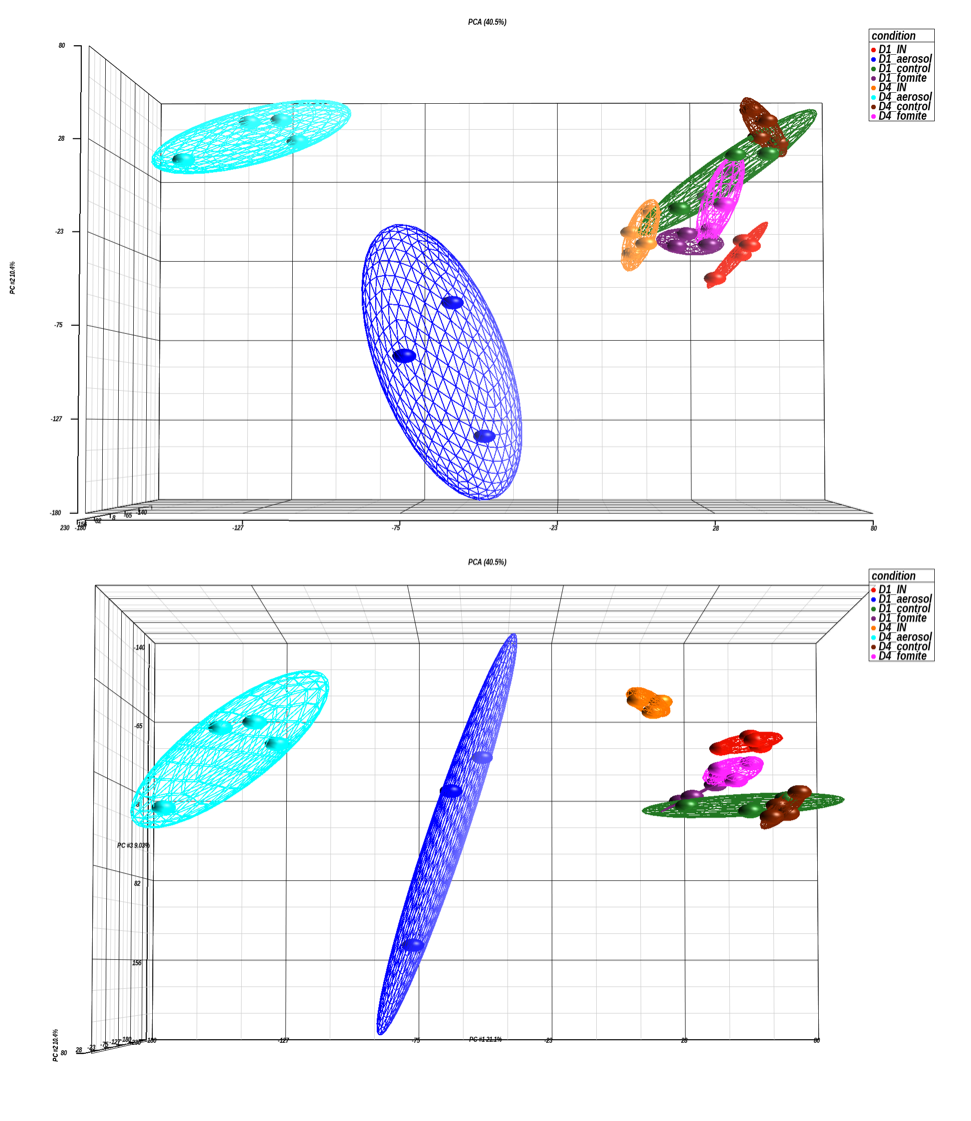
**

**Supplementary Figure 2:** Principal component analysis of lung gene-expression at 1- and 4-days post inoculation. Day and inoculation route is indicated on right.

**Supplementary Figure 3:** Schematic of cage divider.
