# Supplementary material for: SARS-CoV-2 disease severity and transmission efficiency is increased for airborne compared to fomite exposure in Syrian hamsters
Source: Nat Commun. 2021 Aug 17;12:4985. doi: 10.1038/s41467-021-25156-8 (PMC8371001; doi:10.1038/s41467-021-25156-8)
Supplement: Supplementary file 3 — Description of Additional Supplementary Information [file 41467_2021_25156_MOESM3_ESM.docx]

**Supplementary Video 4.** A cage divider was designed, which allowed airflow but no direct contact or fomite transmission between animals. To visualize the airflow when the cage was placed in a normal rodent rack, smoke was generated in the upstream side of the cage and the distribution of the smoke followed until it dispersed.
