# Supplementary material for: SARS-CoV-2 disease severity and transmission efficiency is increased for airborne compared to fomite exposure in Syrian hamsters
Source: Nat Commun. 2021 Aug 17;12:4985. doi: 10.1038/s41467-021-25156-8 (PMC8371001; doi:10.1038/s41467-021-25156-8)
Supplement: Supplementary file 5 — Reporting Summary [file 41467_2021_25156_MOESM5_ESM.pdf]

nature research

Corresponding author(s): Vincent J. Munster

Last updated by author(s): Jun 11, 2021

Reporting Summary

Nature Research wishes to improve the reproducibility of the work that we publish. This form provides structure for consistency and transparency in reporting. For further information on Nature Research policies, see our Editorial Policies and the Editorial Policy Checklist.

Statistics

For all statistical analyses, confirm that the following items are present in the figure legend, table legend, main text, or Methods section.

n/a	Confirmed	
☐	☒	The exact sample size (*n*) for each experimental group/condition, given as a discrete number and unit of measurement
☐	☒	A statement on whether measurements were taken from distinct samples or whether the same sample was measured repeatedly
☐	☒	The statistical test(s) used AND whether they are one- or two-sided<br>*Only common tests should be described solely by name; describe more complex techniques in the Methods section.*
☒	☐	A description of all covariates tested
☒	☐	A description of any assumptions or corrections, such as tests of normality and adjustment for multiple comparisons
☐	☒	A full description of the statistical parameters including central tendency (e.g. means) or other basic estimates (e.g. regression coefficient) AND variation (e.g. standard deviation) or associated estimates of uncertainty (e.g. confidence intervals)
☐	☒	For null hypothesis testing, the test statistic (e.g. *F*, *t*, *r*) with confidence intervals, effect sizes, degrees of freedom and *P* value noted<br>*Give P values as exact values whenever suitable.*
☒	☐	For Bayesian analysis, information on the choice of priors and Markov chain Monte Carlo settings
☒	☐	For hierarchical and complex designs, identification of the appropriate level for tests and full reporting of outcomes
☒	☐	Estimates of effect sizes (e.g. Cohen's *d*, Pearson's *r*), indicating how they were calculated

Our web collection on statistics for biologists contains articles on many of the points above.

Software and code

Policy information about availability of computer code

Data collection	No software was used for data collection
Data analysis	R 4.0.2 using pheatmap and corrplot packages<br>GraphPad Prism 8<br>Ingenuity Pathway Analysis version 01-19-02<br>Partek Genomics Suite

For manuscripts utilizing custom algorithms or software that are central to the research but not yet described in published literature, software must be made available to editors and reviewers. We strongly encourage code deposition in a community repository (e.g. GitHub). See the Nature Research guidelines for submitting code & software for further information.

Data

Policy information about availability of data

All manuscripts must include a data availability statement. This statement should provide the following information, where applicable:

- Accession codes, unique identifiers, or web links for publicly available datasets
- A list of figures that have associated raw data
- A description of any restrictions on data availability

The data generated in this study have been deposited in the figshare data repository (https://doi.org/10.6084/m9.figshare.14642502).
RNA-Seq data was deposited in NCBI GEO under accession number GSE177027 (https://www.ncbi.nlm.nih.gov/geo/query/acc.cgi?acc=GSE177027)

Field-specific reporting

Please select the one below that is the best fit for your research. If you are not sure, read the appropriate sections before making your selection.

☒ Life sciences ☐ Behavioural & social sciences ☐ Ecological, evolutionary & environmental sciences

For a reference copy of the document with all sections, see nature.com/documents/nr-reporting-summary-flat.pdf

Life sciences study design

All studies must disclose on these points even when the disclosure is negative.

Sample size	Power-analysis, animal group size determined to allow statistical significance with 99% CI for assuming 5-fold difference in virus replication
Data exclusions	No data was excluded
Replication	Animal study was done n=4 per group, representing biological replicates.
Randomization	Animals were randomly assigned to groups. All analysis were performed on data collected from these animals and no additional experiments were performed other than animal experiments
Blinding	Data acquisition and analysis were blinded.

Reporting for specific materials, systems and methods

We require information from authors about some types of materials, experimental systems and methods used in many studies. Here, indicate whether each material, system or method listed is relevant to your study. If you are not sure if a list item applies to your research, read the appropriate section before selecting a response.

Materials & experimental systems

n/a	Involved in the study	
☐	☒	Antibodies
☐	☒	Eukaryotic cell lines
☒	☐	Palaeontology and archaeology
☐	☒	Animals and other organisms
☒	☐	Human research participants
☒	☐	Clinical data
☒	☐	Dual use research of concern

Methods

n/a	Involved in the study	
☒	☐	ChIP-seq
☒	☐	Flow cytometry
☒	☐	MRI-based neuroimaging

Antibodies

Antibodies used	KPL Affinity-purified antibody peroxidase-labeled goat-anti-hamster IgGCat.No. 5220-0371 Lot. 10492253 seracare, GenScript U864YFA140-4/CB2093 NP-1, Vector Laboratories ImPress VR anti-rabbit IgG polymer
Validation	Each lot is tested to assure specificity and lot-to-lot consistency using an in-house ELISA assay. Reference number: 14-22-06 (https://www.seracare.com/AntiHamster-IgG-HL-Antibody-PeroxidaseLabeled-5220-0371/)

Eukaryotic cell lines

Policy information about cell lines

Cell line source(s)	VeroE6 UNC, Ralph Baric provided. Also available as VERO C1008 from ATCC (CRL-1586, https://www.atcc.org/products/all/crl-1586.aspx)
Authentication	cells were cytochrome B gene sequenced
Mycoplasma contamination	Tested negative
Commonly misidentified lines (See ICLAC register)	No commonly misidentified cell lines were used in the study.

Animals and other organisms

Policy information about studies involving animals; ARRIVE guidelines recommended for reporting animal research

Laboratory animals	Syrian Golden hamster, female, 4-6 weeks old, Hsd Han AURA
Wild animals	No wild animals were used in study.
Field-collected samples	No field collected samples were used in the study.
Ethics oversight	NIH, NIAID, Rocky Mountain Laboratories ACUC

Note that full information on the approval of the study protocol must also be provided in the manuscript.
